# *In-vitro* Evaluation of Antileishmanial Activity and Toxicity of Artemether with Focus on its Apoptotic Effect 

**Published:** 2013

**Authors:** Parisa Ebrahimisadr, Fatemeh Ghaffarifar, Zuhair Mohammad Hassan

**Affiliations:** a *Department of Parasitology, School of Medical Sciences, Tarbiat Modares University, Tehran, Iran. *; b *Department of Immunology, School of Medical Sciences, Tarbiat Modares University, Tehran, Iran. *

**Keywords:** *Leishmania major*, Promastigotes, Artemether, *In-vitro*, Apoptosis

## Abstract

Artemisinin and its derivatives are very important new class of antimalarial drugs. One of the most important artemisinin derivatives is artemether. The antiparasitic activity of artemether as a derivative of artemisinin is related to endoperoxide bridge in its structure. The aim of this study was the evaluation of antileishmanial effect of artemether, with more focus on its apoptotic effect. In this study we used artemether in concentration of 5, 10, 25, 50 and 100 μg/mL for promastigote assay, promastigote proliferation measurements by MTT assay, detection of apoptotic cells by Flow cytometry analysis and DNA ladder assay. The application of artemether, promastigote IC_50 _was measured as 25 μg/mL. The percentage of apoptotic promastigotes by using 25 μg/mL of artemether was 42.28. The results of present study showed that artemether has inhibition effect on intracellular and extracellular growth of *Leishmania major*. Promastigotes of *Leishmania major *undergo apoptosis after exposure to artemether.

## Introduction

*Leishmania *are protozoan parasites that live either as extracellular promastigotes in phlebotominae insects, or as intracellular amastigotes inside macrophages of mammalian hosts. Parasitic life has to obtain nutrients from their hosts. One of the critical elements is iron. Leishmania spp. parasites require iron for their growth in both the mammalian and the insect stages (1, 2); residing in different environments, each parasite stage likely mobilizes distinct molecules to obtain iron required for replication. The insect stage of the parasite can use different iron sources (1). Chemical drugs for treatment of leishmaniasis include pentavalent antimonate glucantime, pentostam, allopurinol and allopurinol riboside, polyene antibiotics (amphotericin B), aromatic diamidianes, and paramomycin (minosidine) (3). Use of these treatment methods leads to problems such as relapse, drug resistance, adverse drug reaction, secondary bacterial infection, and high costs of treatment (4-5) . A group of researchers for treatment of *Leishmania *have used insects product and medicinal plants, such as Peschiera australis, Peschiera vanherokii, Altharea rosa, Altharea officinalis, Leguminosa faliacaea, Alkanna tinctoria, Pegamum harmala, and Euphorbia mysinites. The plants have inclusive positive results (6-10). 

Artemisinin and its derivatives represent a very important new class of antimalarial drugs; they are becoming more and more commonly used throughout the world. The most important artemisinin derivatives are artesunate, artemether, arteether and dihydroartemisinin. Newer semisynthetic and synthetic derivatives are also being developed. The artemisinin derivatives act quickly and are eliminated quickly. The antiparasitic activity of artemisinin and its derivative are related to endoperoxide bridge in its structure. Artemether is one of the new, promising semi- synthetic anti-malarial drugs (sesquiterpene lactone endoperoxides) derived from the natural product artimisinin, extracted from the plant *Artemisia annua*. It is used for the treatment of erythro- cytic stages of chloroquine-resistant *Plasmodium falciparum *and cerebral malaria (11).

**Figure 1:**
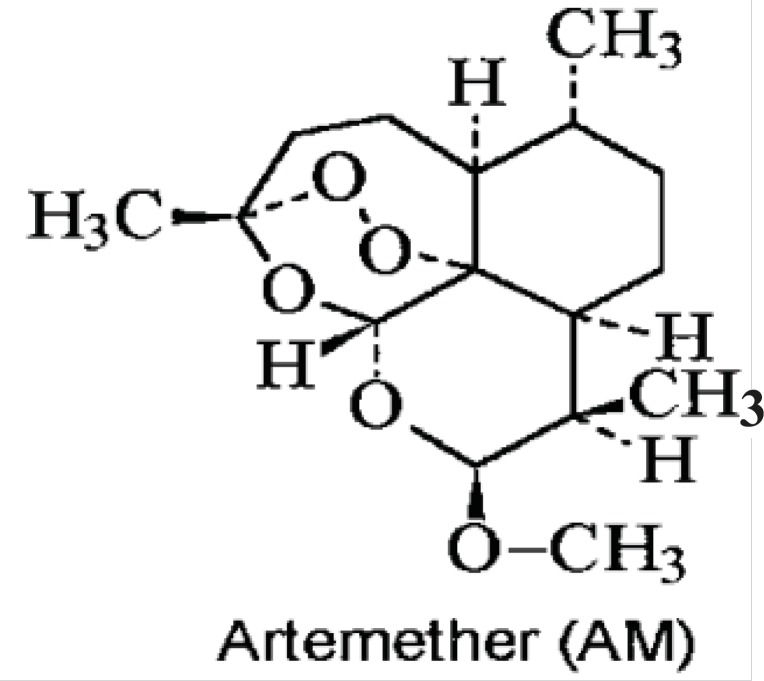
Chemical structure of artemether

The Chemical structure of artemether has been shown in Figure1. In this study the antileishmanial properties of artemether, with more focus on its apoptotic effect, have been evaluated *in-vitro*.

## Experimental

*Leishmania culture*

*L. major *(MRHO/IR/75/ER) was cultured in RPMI 1640 (Gibco) with 20% FCS (Gibco) for preparation of adequate promastigotes.

*Artemether preparation*

Artemether was obtained from Exim Pharm Co. US. Stock solutions of artemether were freshly prepared in ethanol–water (1:1, v/v). At first 1 mg of artemether was dissolved in 0.5 mL ethanol and then slowly diluted with 0.5 mL water. For better dissolving, we used 100 μL of ethanol and 100 μL of water respectively to get the volume of 1 mL (12) .

*Promastigote assay*

Promastigote assay was carried out using a previously described direct counting assay based on growth inhibition (13). The effects of the crude extracts were evaluated in 24-well microtitre plates. The promastigotes were seeded at an initial concentration equivalent to that of early log phase (2 × 10^5 ^promastigotes/mL). Results were expressed as the concentration that inhibited parasite growth by 50% (IC_50_). IC_50_ was measured by calculating from log-probit analyses using linear regression. 

*Promastigote proliferation measurements by MTT [3-(4, 5-methylthiazol-2-yl)-2, 5-diphenyltetrazolium bromide] assay*

In 3 separate 96-well microtitre plates, 3×10^6^ promastigotes of *Leishmania major *per well were cultured in RPMI 1640 (Gibco) and 20% FCS (Gibco) and allowed to multiply for 24,48 and 72 h in the medium alone (control group), in solvent (other control group) or in the presence of artemether in concentration of 5,10, 25, 50 and 100 μg/mL. After these times 20 μL of tetrazolium (Roche, Germany) (5 mg/mL) was added to each well and encubated in 18 °C for 4 h then centrifuged in 1000g for 10 min, the supernatant was discarded and 100 μL of DMSO was added to each wells and resuspended. The OD was read by ELISA reader in 540 nm. (14).

*Amastigote/ Macrophage Assay*

Drug susceptibilities of the amastigotes in the macrophage BALB/c mice were determined by following a modification of the method of Chang. Briefly, peritoneal macrophages were collected and infected *in-vitro *with promastigotes in RPMI medium with 10% heat-inactivated fetal bovine serum to yield 1×10^6^ cells and 1×10^7^ promastigotes per ml. The cultures were incubated for about 3 days at 37 °C in 5% CO_2_ to allow phagocytosis of the promastigotes and adhesion to the surface. 

The number of parasites were calculated in 100 macrophages, then 2 ×10^5^ macrophages/well were cultured in 24-well plates with RPMI and 10% FBS. The extracts of plant with the concentration that obtained by IC_50_, were added to the wells after 24 h. The plates incubated in 37 °C and 5% CO_2_ incubator and the amastigotes in 100 macrophages were counted in 24, 48 and 60 h after incubation (15).

*Flow cytometry analysis*

The Annexin-V Staining Kit (Roche, Germany) with propidium iodide (PI) was used for the detection of apoptotic and necrotic cells according to the manufacturer›s protocol. Briefly, promastigotes were washed in cold phosphate-buffered saline (PBS) by centrifugation at 1400 g for 10 min and the pellet resuspendeed in binding buffer to a concentration of 1 × 10^6^/mL of promastigotes. Then, they were incubated for 15 min in dark and at room temperature in 10 μL of Annexin-V in the presence of PI. Then, the samples were analyzed with FACSC alibur flow cytometer (Becton Dickinson and Cell Quest software), and the percentage of positive cells was determined for each sample.

*DNA ladder assay*

An apoptotic DNA ladder kit (Roche, Germany) was used to extract DNA from apoptosis-induced cells according to the manufacturer›s protocol. Briefly, promastigotes (5 × 10^6^ cells) were incubated alone or with 10, 25, and 50 μg/mL of artemether for 24 h, then collected and froze in -20 °C. An apoptotic DNA ladder kit was used to extract DNA from apoptosis-induced promastigotes. DNA was electrophoresed in 1 % agarose gels at 100 V for 2 h. The analysis of DNA fragmentation carried on manufacturer›s apoptotic DNA ladder kit.

## Results

*Anti-leishmanial activity of artemether*

**Figure2:**
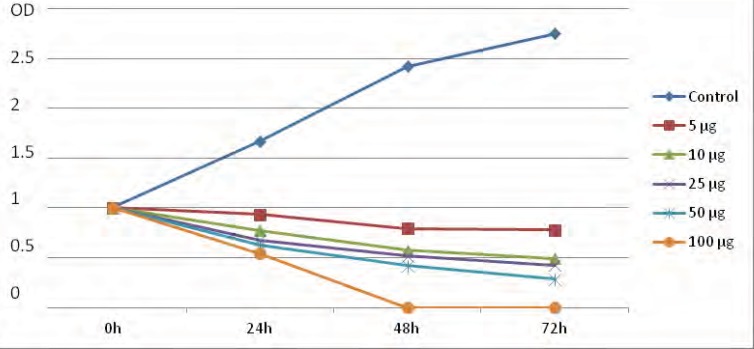
The OD in 540 nm obtained from MTT assay of *Leishmania major *promastigotes in the presence of various concentrations of artemether in comparison with control group in 0, 24, 48 and 72 h

Following the application of artemether, promastigote IC_50_ were measured as 25 μg/mL. Cytotoxic potential of artemether on *L. major *promastigotes was measured by using the MTT assay. Grouth inhibition of promastigotes was evaluated and the percentage of *Leishmania major *promastigotes in the presence of various concentrations of artemether in comparison with control group in 0, 24, 48 and 72 h are showed in Figure 2.

*Flow cytometric analysis*

**Figure 3:**
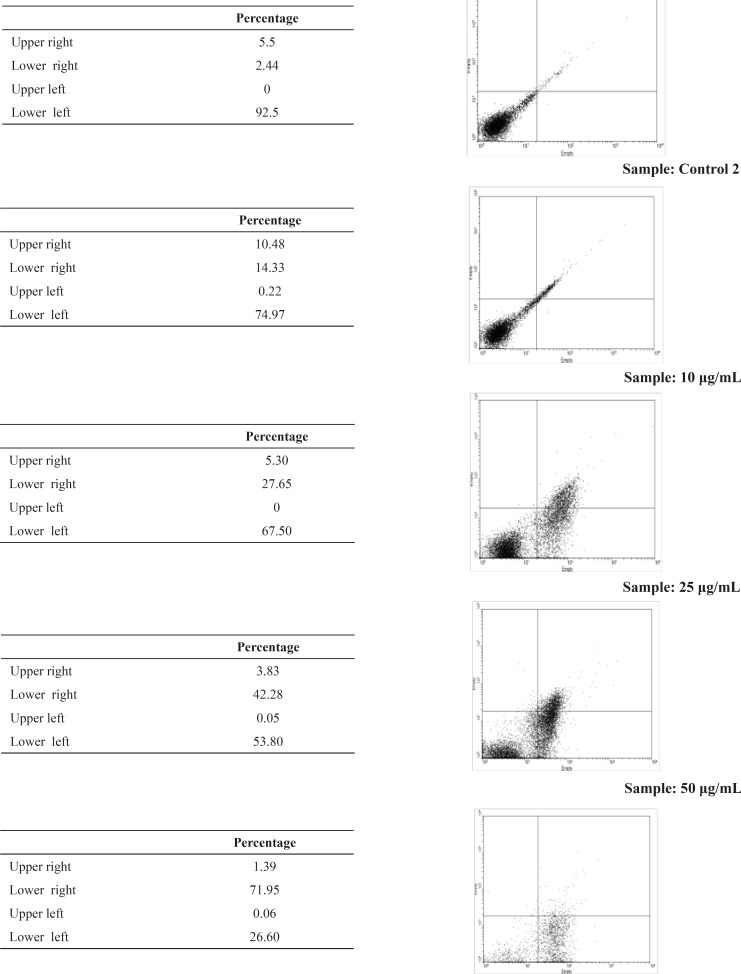
Flow cytometry analysis of promastigotes following treatment with artemether and after labeling with annexin-V and PI. Control 1, without treatinh. Control 2, with ethanol as solvent. Samples 10 μg/mL, 25 μg/mL and 50 μg/mL are samples that treated with 10 μg/mL, 25 μg/mL and 50 μg/mL of artemether respectively. Lower right region (LR) belongs to apoptotic cells (annexin positive) and upper left region (UL) belongs to necrotic cells (PI positive).

Flow cytometric analysis was performed after labeling with Annexin-V FLUOS and the percentages of viable, necrotic and apoptotic cells were determined for each concentration of artemether. Fluorescein-conjugated Annexin-V is used to detect the externalized phosphatidylserine as it has a high binding affinity to this phospholipid component. Additionally, annexin-V FLUOS allows distinguishing between apoptotic cells, necrotic cells, and surviving cells. The percentage of apoptotic promastigotes by using 0, 25 and 50 μg/mL of artemether was 2.44, 42.28 and 71.95μg/mL respectively (Figure 3).

*Amastigote-macrophage assay*

The mean of the amastigotes/ macrophages before adding the extracts was 0.98 and after adding artemether with 5, 10, 25, 50 and 100 μg/mL after 72 h was 0.78, 0.64, 0.49, 0.30 and 0.21 respectively whereas it was 1.6 for control group Table 1.

**Table 1:** Mean and SD of amastigote/macrophage in 72 h after adding artemether with different concentrations

**Artemether concentration**	**Control 1**	**Control 2**	**5 μg/mL**	**10 μg/mL**	**25 μg/mL**	**50 μg/mL**	**100 μg/mL**
Mean , SD of amastigote/macrophage	o.98, 0.08	0.92, 0.11	0.78, 0.06	0.64, 0.05	0.49, 0.05	0.30, 0.3	0.21, 0.02

*DNA ladder assay*

DNA fragmentation of promastogotes of Leishmania major was confirmed by presence of DNA fragments in comparison with standard fragmented DNA. The fragments size was about 120, 300, 800, 900 and 1050 bp (Figure 3).

## Discussion

The drug of choice for treatment of cutaneous leishmaniasis is Glucantime and Pentostam. Both of them have toxic side effects. Pharmaceutical research represents a major strategy for discovering new drugs with minimal toxicity.

Adverse effects related to artemisinin derivatives are rare (16). 

Artemisinin derivatives also appear to be safe for pregnant women (17).

In 1991, Meshnick *et al*. showed that artemisinin interacted with intraparasitic heme, and suggested that intraparasitic heme or iron might function to activate artemisinin inside the parasite into toxic free radicals (18).

Artemether that is a derivative of artemisinin, have been shown to exhibit antileishmanial effects. Significant progress has been made with artemether, is due to the methyl ether derivative of artemisinin**. **Artemether with high efficacy and low toxicity could be a suitable candidate for treatment of leishmaniasis.

In the presence of iron, artemisinin and its derivatives are activated, produce free radicals and cause cell death. 

Artemisinin and its derivatives are approved for treatment of drug resistant malaria and have cytotoxic effects against some cancer cell lines such as leukemia, colon cancer, melanoma and breast cancer.

Artemether is effective in treatment of parasitic diseases including Schistosoma(treatment of Schistosoma japonicum,mansoni,hematobiuom were done alternatively 2,3 and 4 weeks) (19) ,Clonorchis (20), Fasciola (21), and malaria (22).

In this study we showed that artemether had apoptotic effect on Leishmania major promastigotes, and could inhibit the growth of both of promastigotes and amastigotes *in-vitro*.

The results of present study showed that artemether can be applied as new treatment for cutaneous leishmaniasis.

Induction of apoptosis is one of advantages of artemether against *Leishmania major. *This results indicated that Promastigotes of *Leishmania major *undergo apoptosis after exposure to artemether. The other prominent feature observed in this study was DNA laddering that confirms the apoptosis too.

**Figure 4:**
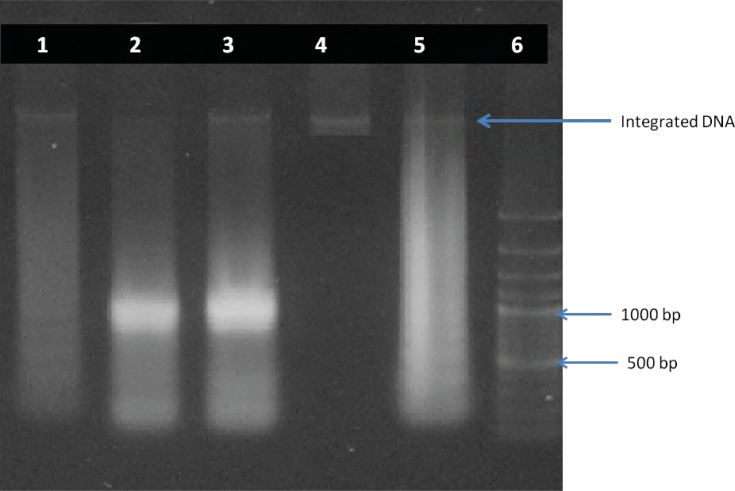
DNA fragmentation analysis by agarose gel electrophoresis. The DNA of promastigotes treated with 10, 25 and 50 μg of artemether after 24 h of incubation (lines 1-3). Integrated DNA (line4). Standard apoptotic ladder (line 5) and DNA marker (line 6).
